# Editorial: Molecular and cellular aspects of regulatory and subjacent mechanisms in host/microbiota association and its involvement in cancer

**DOI:** 10.3389/fcell.2022.989208

**Published:** 2022-08-29

**Authors:** Ana I. Álvarez-Mercado, María José Sáez-Lara, Julio Plaza-Diaz

**Affiliations:** ^1^ Instituto de Investigación Biosanitaria IBS.GRANADA, Complejo Hospitalario Universitario de Granada, Granada, Spain; ^2^ Department of Biochemistry and Molecular Biology II, School of Pharmacy, University of Granada, Granada, Spain; ^3^ Institute of Nutrition and Food Technology, Biomedical Research Center, University of Granada, Granada, Armilla, Spain; ^4^ Department of Biochemistry and Molecular Biology I, School of Sciences, University of Granada, Granada, Spain; ^5^ Children’s Hospital of Eastern Ontario Research Institute, Ottawa, ON, Canada

**Keywords:** microbiota, dysbiosis, cancer, host, homeostais

## Gut microbiota and cancer

Microbiota can be defined as the assemblage of microbes in a specific environment, while microbiomes are the genetic composition of those microbes (Berg et al., 2020). The human microbiota is composed of symbiotic microbial cells, primarily bacteria in their gut. Regulation of the microbiota is mediated by microbiological, host characteristics, dietary patterns, and environmental factors (Ursell et al., 2012).

Human health is maintained by a dynamic equilibrium between the microbiota and the host. It plays a role in both local and remote aspects of fundamental processes such as inflammation and immune responses. There is evidence that microbiota is capable of producing metabolites that protect host homeostasis, however, under certain conditions, those microbes may be detrimental (Kho and Lal, 2018). In recent studies, the gut microbiota has been implicated in both the preservation of host health and the development of various pathologies including cancer (Vivarelli et al., 2019).

Cancer involves a number of diseases in which abnormal cells divide without control and can invade nearby tissues (Akbarian et al., 2022). Approximately 10 million people worldwide will die from cancer in 2020, which represents nearly one out of every six deaths (Ferlay et al., 2020). Evidence is emerging that the gut microbiota can be controlled in order to improve the treatment of cancer. Microbes can be used as adjuvants for checkpoint immunotherapy to enhance cancer care (Bhatt et al., 2017).

A variety of bacteria in the gut have been shown to create DNA-damaging toxins and carcinogenic metabolites, to stimulate cancer-promoting inflammation, to make tumors resistant to chemotherapy drugs, and to suppress the body’s immune response to cancer (Kartikasari et al., 2021). Despite chemotherapy’s devastating effects on microbial diversity and resulting changes in gut homeostasis and severe side effects, the microbiota may contribute to the heterogeneous response of patients to treatment. Those changes in the gut microbiota can affect oncogenesis, tumor progression, and even the response to cancer therapies. Host defense against pathogens is mediated by the gut microbiota and requires an understanding of the current microenvironment and a distinction between commensal and occasional bacteria (Deleemans et al., 2019).

## Regulatory and subjacent mechanisms in a host-microbiota relationship and its involvement in cancer

The special issue of Frontiers in Cell and Developmental Biology contains six articles that provide an overview of recent advances in the study of molecular and cellular aspects of regulatory mechanisms underlying host/microbiota mutualism as well as some microbial signatures and disease related to microbiota signature.

Accordingly, Zhou et al. investigate the relationship between commensal microbes, inflammation, and the risk of cancer development. Researchers have observed a cross-talk between commensal microbes and colonized mucosa, suggesting the possibility of anergic inflammation in nasopharyngeal carcinoma cells despite widespread constitutive activation of NF-κB. The presence of oxidative stress is another factor that plays a dominant role in inflammatory-related diseases (including cancer). There is increasing evidence that oxidative stress and gut microbiota are closely related. As a result of this research, promising gut microbiome/redox-targeted therapeutic strategies may be developed, along with a deeper understanding of the role that oxidative stress and gut microbiome play during the progression of cancer, as described in Ni et al.

Other researchers examine microbiota signatures in specific tissues and diseases related to microbiota signatures. According to Li et al. key genera may contribute to the topographic variances in the microbiota of tumor-bearing colorectums. In their study, Liu X. et al. reported that *Lactobacillus* spp. were representative of the asymptomatic controls, while the *Prevotella* spp. were found in the vulvar lichen sclerous group. As described by Chen et al. functionally active microorganisms play an essential role in the occurrence and migration of endometrial cancer. Liu J. et al. have revised the gut bacterial composition associated with autism spectrum disorders as well as the possible mechanism underlying the disease onset and progression. Consequently, there are numerous challenges and potential solutions that need to be addressed. Among these challenges are the development of effective and even tailored therapies for cancer as well as the modulation of the gut microbiota.

In summary, the six contributions to this special issue contribute to understanding the relationship between the presence of certain microbes and the risk and progression of cancer, and also the existence of specific signatures for different types of tissues or diseases related to microbiota signatures and their manipulation to improve the health of cancer patients. In order to achieve more personalized and precision health in cancer care, strategies for maintaining a balanced microbiota (dietary interventions, microbes administration, or fecal microbiota transplantation) should be further explored.
